# Wrist fractures and their impact in daily living functionality on elderly people: a prospective cohort study

**DOI:** 10.1186/s12877-015-0176-z

**Published:** 2016-01-14

**Authors:** Itziar Vergara, Kalliopi Vrotsou, Miren Orive, Susana Garcia-Gutierrez, Nerea Gonzalez, Carlota Las Hayas, Jose M. Quintana

**Affiliations:** Unidad de investigacion AP-OSIS Gipuzkoa, Osakidetza, Red de Investigación en Servicios Sanitarios y Enfermedades Crónicas (REDISSEC), Instituto de Investigación Sanitaria Biodonostia, San Sebastian-Donostia, Spain; Unidad de Investigación, Hospital Galdakao-Usansolo, Red de Investigación en Servicios Sanitarios y Enfermedades Crónicas (REDISSEC), Galdakao, Bizkaia Spain; Psychology Department of Personality, Assessment and Treatment, University of Deusto, Bilbao, Bizkaia Spain; Red de Investigación en Servicios Sanitarios y Enfermedades Crónicas (REDISSEC), Galdakao, Bizkaia Spain

**Keywords:** Wrist fractures, Cohort study, Prospective study, Elderly

## Abstract

**Background:**

Wrist fractures are the most common arm fractures in older adults. The impact of wrist fractures on daily functionality has been less studied than that of other types and so, less is known about the complexity of factors related to the functional impact of these fractures. This study is aimed to assess the role of individual and health care factors and its association with daily living functional changes after a wrist fracture.

**Methods:**

A prospective cohort of patients aged 65 or more, affected by a fracture due to a fall, was conducted. These patients were identified at the emergency rooms of the six participating hospitals. As independent factors, the following were studied: socio-demographic data, characteristics of the fracture, health-related quality of life, wrist function and provided treatment. The main outcome was functional status measured by the Barthel Index for daily living basic activities and the Lawton Instrumental Activities of Daily Living (IADL) Scale for daily living instrumental activities. Data were collected at baseline just after the fall and after six months of follow-up. Patients were considered to have deteriorated if their functional status as measured by Barthel Index or Lawton IADL scores decreased in a significant way during the six months of follow up.

**Results:**

Barthel Index and/or Lawton IADL scores fell at six months after the fracture in 33 % of participants. This functional decline was more frequent in patients with comorbidity (*p* < 0.0001), polypharmacy (*p* < 0.0001), low health-related quality of life prior to the fall (*p* < 0.0001) and lower educational level (*p* = 0.009). The derived multivariate models show that patients that become dependent six months after the fall, have advanced age, severe chronic diseases, low functional performance prior to the fracture, and repeated episodes of accidental falls. This profile is consistent with a frailty phenotype.

**Conclusions:**

Wrist fractures are associated to the occurrence of dependence, especially in frail patients. These patients could benefit from being identified at the time the fracture is treated, in order to tackle their complex needs and so, prevent some of the burden of dependence generated by these fractures.

## Background

Wrist fractures are the most common arm fractures in older adults. Their incidence varies from 2.4 to 10 per 1000 people per year according to published studies [1, 2]. The most frequent profile of patients affected by a wrist fracture is a woman younger than 75, healthy and functionally independent [3].

Although the expected impact on overall functionality of wrist fractures may not be as devastating as that of hip or vertebral fractures, considering that hands are fundamental for performing basic and instrumental daily activities, some degree of functional affectation should be expected. This affectation has been less studied than that of hip fractures [3]. Existing evidence suggests an association between fracture type and received treatment with further functional prognosis [4–8]. Nevertheless, less is known about the complexity of other factors (patient and treatment related) actually affecting the functional impact of these fractures.

This study aimed to assess the role of individual (i.e. sex, age, daily living functional status) and health care factors and their association with daily living functional changes after a wrist fracture.

## Methods

A prospective six-month follow-up cohort study was conducted. Patients aged 65 years and older affected by a wrist fracture (ICD-9 codes 813.4 and 813.5) due to an accidental fall were included in the study. All patients were informed about the characteristics of the study and provided informed consent. These patients were identified and recruited at the emergency rooms (ER) where they sought medical attention after the fall. Six ER located in six public hospitals of the Basque Health Service (Osakidetza) provided patients. The population covered by each hospital as well as the type of services and the level of performance delivered at them, were comparable. The Ethics Committee of the Galdakao-Usansolo Hospital approved the study.

Patients whose fall was due to a previous syncope episode [5], those for whom a pathologic fracture was suspected, those with additional fractures as well as those with cognitive impairment, were excluded. Patients who completed less than 50 % of the questionnaires were considered lost to follow-up.

Information was collected by two trained psychologist, member of the research team at baseline, during the first week after the fall, and after six months. Interviews lasted approximately 40 minutes. Psychologists had been previously trained on interview skills and the battery of tests. Baseline information was obtained from different sources and included: from the ER medical record, socio-demographic data, characteristics of the fracture, diagnostic tests performed, proposed treatment, and destination at discharge; from the hospital medical records, comorbidity (Charlson comorbidity Index and all individual pathologies composing the latter), treatment of the fracture (conservative vs. surgery), hospital admission (length of stay, complications), and destination at discharge (home, residence, long term hospital), or date of death; from the personal interview, patient reported outcomes (PRO): social support network, level of education, income, self-reported health-related quality of life (HRQoL) and functionality (Barthel Index and Lawton IADL Scale, Quick Dash). These three were explored retrospectively capturing functionality status before the fall.

Six months after the fall patients were assessed again by reviewing the clinical records and by the completion of the same battery of questionnaires used at baseline. Questionnaires were sent to all the participants by postal mail. If the material was not returned, a reminder letter was sent at 21 and again at 35 days. If still no reply received, patients were telephoned to increase the response rate and the interview was performed by the same trained interviewer via the phone, when possible. Some further details of the methodology as well as a full description of the questionnaires used at this study can be found in a related article [6]. The current data is part of a bigger study aimed to describe the treatment provided to elder patients presenting hip or wrist fractures due to accidental falls. The methodology of the overall project was described in the referred previous article were the results regarding hip fractures were presented.

Functional capacity was measured with the Barthel Index [7, 8] and the Lawton Scale [9, 10] to assess the ability to perform basic activities of daily living (BADL) and instrumental activities of daily living (IADL) respectively. These two are the main outcomes studied.

Deterioration (yes/no) in BADL and IADL was defined and studied separately. Regarding BADL, patients were considered as deteriorated if their post-fall Barthel scores were <90 points or they had decreased by more than 10 % compared to baseline, given that 90 points is defined as a threshold for moderate dependency and that a 10 % decrease may imply in some cases, a change in the level of independence [11]. Regarding IADL, Lawton post-fall values of <5 points or a decrease of 2 points were considered to indicate deterioration, taking into account the responsiveness of this test [10].

Wrist function was assessed with the specific questionnaire QuickDASH. It consists of 11 items derived from the disabilities of the arm, shoulder and hand (DASH) questionnaire [12, 13]. It has been shown to be as valid and reliable as the full DASH [14]. It assesses upper extremity symptoms (pain, weakness) and function. The score is scaled between 0 and 100, with higher scores indicating worse upper-extremity function [15].

HRQoL was also evaluated, with the 12-Item Short Form Health Survey (SF-12) [16, 17]. It is a generic instrument that contains 12 items from the SF-36 Health Survey [18] and reproduces both, the physical component summary score (PCS) and the mental component summary (MCS) score.

### Statistical analysis

Categorical data are presented as frequencies with percentages (%) and continuous data as means with standard deviations (SD). Associations between categorical variables were assessed with the chi-square test. The two-sample t test and Mann-Whitney test were implemented for two-group comparisons of continuous variables depending on their distribution. P-values <0.05 were considered statistically significant. Patient-reported outcome (PRO) measures were examined both as baseline values and as pre-post differences. To ensure that negative values indicate deterioration, differences were calculated as post-pre values for most PRO measures. Quick DASH differences were derived as pre-post values for the same reason.

Univariate and multivariate logistic regression models were fitted. The multivariate regression model was constructed with backward selection, initially considering all variables with p-values ≤0.10 in the univariate stage. Charlson index and the most frequent pathologies composing the latter were studied in separate models. Regression model results are presented as odds ratios (ORs) and 95 % confidence intervals (CIs). Estimates related to SF-12 and QuickDASH correspond to 10-unit score increases, with the respective score values transformed accordingly prior to model fitting. The correlation matrix of the estimated effects, their eigenvalues and proportion of variation were tested for assessing collinearity presence [19]. The performance of the fitted models was assessed by examining the deviance residuals, the Hosmer-Lemeshow test, the R-square values and the area under the curve (AUC). All analyses were performed with the SAS software version 9.3.

## Results

**Fig. 1:**
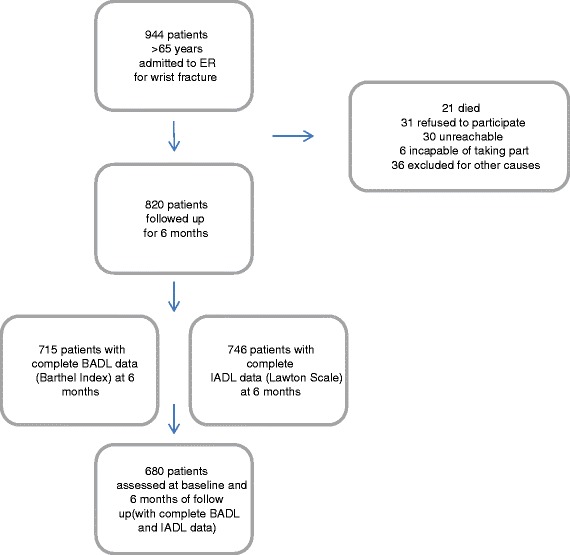
Flow chart of the recruitment and follow-up process. Flow chart representing the number of patients at recruitment, baseline and the 6-month follow up. Causes of loss to follow-up, and completion of functional status assessments are included

Overall, 944 patients were initially recruited at the ER services of the participating hospitals for a wrist fracture due to an accidental fall. From these, 680 subjects fulfilled the inclusion criteria and were included in the study and followed up for six months (Fig. 1).

The study participants were mostly women (89 %), with a mean age of 76.5 years (SD: 7.0). From a health status perspective, they had a high degree of comorbidity with 66 % of participants presenting a Charlson Index greater than or equal to 2. The most prevalent diseases were diabetes, present in 11 % of patients, chronic bronchitis or COPD in 10 % and cardiovascular disease in 9 % of the studied patients. Only 12 % of the patients were not taking any prescription drugs, and 23 % were taking four or more. From a social perspective, 70 % were living with a family member (spouse, mostly) and only 10 % were receiving some kind of institutional social support. Most patients (90 %) were able to read and write or had completed primary education.

Studied fractures were mostly extra-articular (86 %) and were managed conservatively (84 %). Administered treatment was related to the type of fracture and also significantly related to age, with younger participants being more likely to receive surgical treatment (*p* < 0.0001). Only 25 % of the patients were referred for rehabilitation treatment, receiving a mean of 15.6 sessions (SD: 19.8). The decision to refer was related to: the type of fracture, being more frequent for articular fractures (*p* = 0.001); to age, younger patients being more likely to receive rehabilitation (*p* = 0.007); and to gender, with women receiving this kind of treatment more frequently (*p* = 0.008).

**Fig. 2:**
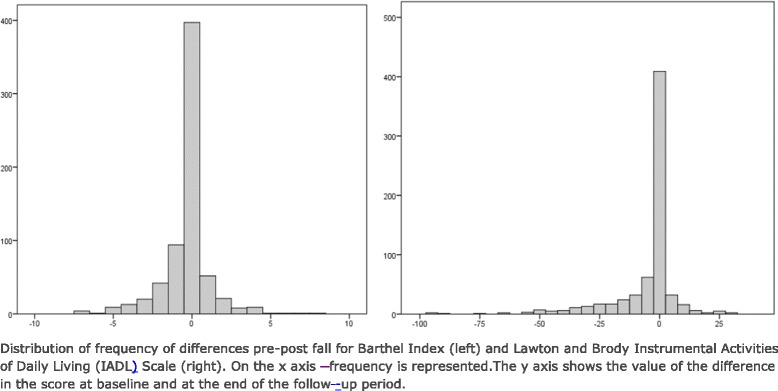
Difference pre-post in Barthel Index and Lawton and Brody IADL Scale

Considering basic functional performance, participants were generally independent (mean 95.6, SD: 12.2). After six months of follow-up, BADL functional performance showed a notable decrease with 24 % of the subjects obtaining lower scores on the Barthel Index. Regarding Instrumental functional performance patients were independent at baseline (mean 6.7, SD: 2.2), but after six months of follow-up 23 % of them obtained lower scores on Lawton IADL Scale. Pre post differences are shown in Fig. 2.

Baseline characteristics of the studied participants according their functional status at six months, in relation to BADL and IADL, are presented in Tables 1 and 2.

**Table 1:** Baseline characteristics of the sample

Variable	Total (*n* = 680)
Age; mean(SD)	76.5 (7.0)
Sex	
Female	603 (89)
Charlson Index	
0	78 (12)
1	144 (22)
≥2	441 (66)
Cardiovascular disease	
yes	61 (9)
Lung disease	
yes	67 (10)
Diabetes without complications	
yes	72 (11)
Cerebrovascular disease	
yes	37 (5)
Educational level	
Illiterate	13 (2)
Able to read & write	315 (47)
Primary education	268 (40)
Secondary education	54 (8)
University qualifications	22 (3)
Way of living	
With a relative	473 (70)
Along, receiving help	158 (24)
Along	42 (6)
Current medication use	
None	82 (12)
1-3 medications	440 (65)
≥4 medications	155 (23)
Previous Falls	
No	492 (73)
Yes, with fracture	28 (4)

Values in cells are frequency (percentage) unless otherwise stated. For variables with missing data frequencies do not add up to N. SD: standard deviation. For binary variables only one category is presented

**Table 2:** Baseline characteristics of the sample as a function of subsequent deterioration in ability to perform any of BADL and IADL

Variable	BADL performance			IADL performance		
	No Deterioration (*n* = 508)	Deterioration (*n* = 172)	*p*-value	No Deterioration (*n* = 510)	Deterioration (*n* = 170)	*p*-value
Age; mean(SD)	74.9(6.3)	81.3 (6.8)	<0.0001	74.8 (6.3)	81.6 (6.5)	<0.0001
Sex						
Female	450 (99)	153 (99)	0.895	460 (90)	143 (84)	0.030
Charlson Index						
0	68 (14)	10 (6)	<0.0001	68 (13)	10 (6)	<0.0001
1	123 (25)	21 (13)		124 (25)	20 (12)	
≥2	306 (61)	135 (81)		308 (62)	133 (82)	
Current medication use						
None	73 (15)	9 (5)	<0.0001	75 (15)	7 (4)	<0.0001
1-3 medications	341 (67)	99 (58)		340 (67)	100 (59)	
≥4 medications	92 (18)	63 (37)		93 (18)	62 (37)	
Educational level						
Illiterate	7 (2)	6 (3)	0.009	7 (1)	6 (4)	0.017
Able to read & write	225 (45)	90 (54)		227 (45)	88 (53)	
Primary education	205 (40)	63 (37)		205 (41)	63 (38)	
Secondary education	46 (9)	8 (5)		48 (9)	6 (4)	
University qualifications	21 (4)	1 (1)		19 (4)	3 (1)	
Previous Falls						
No	385 (77)	107 (63)	<0.0001	386 (76)	106 (63)	0.003
Yes without fractures	105 (21)	47 (27)		99 (20)	53 (32)	
Yes, with fracture	11 (2)	17 (10)		19 (4)	9 (5)	
Performed treatment						
Surgery	91 (18)	18 (11)	0.022	94 (19)	15 (9)	0.003
Referral for rehabilitation						
No	369 (73)	136 (80)	0.066	365 (72)	140(83)	0.003
Patient-reported measures of HRQoL and functionality: mean (SD)						
SF-12: PCS						
Pre fall	49.2 (8.6)	39.1 (10.9)	<0.0001	48.7 (9.1)	40.7 (11.1)	<0.0001
Difference (post-pre)	−6.9 (10.6)	−9.8 (11.1)	0.007	−7.3 (10.7)	−8.7 (11.1)	0.173
SF-12: MCS						
Pre fall	52.8 (8.5)	48.6 (11.0)	<0.0001	52.9 (8.6)	48.5 (10.6)	<0.0001
Difference (post-pre)	−0.9 (10.8)	−4.5 (13.0)	0.003	−1.1 (10.8)	−3.8 (13.2)	0.031
Quick DASH						
Pre fall	5.8 (10.7)	20.6 (20.2)	<0.0001	6.3 (11.0)	19.3 (20.8)	<0.0001
Difference (pre-post)	−18.7 (22.1)	−29.9 (27.3)	<0.0001	−19.7 (22.5)	−26.9 (27.4)	0.002
Lawton						
Pre Fall	7.4 (1.4)	4.7 (2.8)	<0.0001	-	-	
Difference (post-pre)	−0.03 (1.16)	−1.0 (2.1)	<0.0001	-	-	
Barthel						
Pre fall	-	-		99 (5)	87 (20)	<0.0001
Difference (post-pre)	-	--		−1.0 (5.9)	−17.0 (22.1)	<0.0001

Values in cells are frequency (percentage) unless otherwise stated. For variables with missing data frequencies do not add up to N. SD: standard deviation. Basic activities of daily living (BADL) were assessed through the Barthel Index; BADL was considered to have deteriorated if post-fall scores were <90 points or a pre-post score decrease of more than 10 % has occurrred. Instrumental activities of daily living (IADL) were assessed by the Lawton Scale; IADL was considered to have deteriorated when post-fall scores of <5 points were observed or a pre-post score decrease of 2 points occurred. For binary variables only one category is presented

A set of factors were systematically associated with decreases in both BADL and IADL functionality. From the patient´s medical perspective, these were: age, comorbidity, the presence of severe chronic diseases (cardiovascular, COPD, diabetes or dementia), polypharmacy, previous falls, baseline function and both generic and specific HRQoL. Further, associations were found with two factors related to social and living conditions: low educational level and receiving some kind of social service, and one factor related to health service provision: the type of treatment performed, specifically surgical treatment. Additionally, decreases in IADL functionality were associated with sex, specifically masculine, and the referral for rehabilitation treatment.

Multivariate models assessing the simultaneous impact of various factors on functional progression are presented for BADL and for IADL performance (Table 3). After adjusting for age and sex, HRQoL as reflected by the SF-12 MCS and PCS and baseline wrist function measured by QuickDASH, were present in both models.

**Table 3:** Multivariate logistic regression models for BADL and IADL assessment at six months

	BADL model		IADL model	
Variable	OR (95 % CI)	*p*-value	OR (95 % CI)	*p*-value
Age	1.12 (1.09, 1.16)	<0.0001	1.15 (1.11, 1.19)	<0.0001
Sex				
Male	Ref.	-	Ref.	-
Female	0.91 (0.46, 1.78)	0.771	0.37 (0.20, 0.69)	0.002
Cardiovascular disease				
No	Ref.	-	-	-
Yes	2.35 (1.20, 4.63)	0.013	-	-
Baseline HRQoL				
SF-12 PCS	0.51 (0.40, 0.66)	<0.0001	0.66 (0.51, 0.84)	0.001
SF-12 MCS	0.63 (0.50, 0.79)	<0.0001	0.60 (0.48, 0.74)	<0.0001
QuickDASH	1.24 (1.04, 1.48)	0.016	1.25 (1.05, 1.47)	0.011
Previous Falls				
No	Ref.	-	-	-
Yes without fractures	0.91 (0.54, 1.53)	0.723		
Yes with fractures	3.09 (1.06, 8.99)	0.038	-	-
Goodness–of-fit statistics				
Hosmer-Lemeshow	*p* = 0.651		*p* = 0.439	
R square / Adjusted R square	0.285/0.420		0.260/0.385	
AUC	0.847		0.839	

*OR* Odds Ratio, *95 % CI* 95 % Confidence Interval. BADL model: multivariate model considering status at six months (deteriorated or not) based on ability to perform basic activities of daily living (BADL) as assessed assessed through the Barthel Index; BADL was considered to have deteriorated if post-fall scores were <90 points or a pre-post score decrease of more than 10 % has occurrred. IADL model: multivariate model considering status at six months based on ability to perform instrumental activities of daily living (IADL) as assessed by the Lawton Scale; IADL was considered to have deteriorated when post-fall scores of <5 points were observed or a pre-post score decrease of 2 points occurred. Estimates presented: for age refer to 1-unit increases; and for baseline health-related quality of life (HRQoL) refer to 10-unit increases in the respective score scales. SF-12 PCS (SF-12 physical component summary); SF-12 MCS (SF-12 mental component summary); AUC (area under the curve)

According to these models, older patients with cardiovascular disease and a history of fractures causing falls had higher odds of BADL functional deterioration six months after an accidental fall. On the other hand, higher baseline HRQoL scores, both in physical and mental components, were associated with a lower probability of having a reduced ability to perform BADL and IADL six months after the fracture. No collinearity was diagnosed among the variables included in the models. Both models presented R-square values ≥26 %, and AUC around 0.84.

## Discussion

Patients sustaining wrist fractures are mostly women, of advanced age but independent in BADL and IADL prior to the fracture. This profile of patients is markedly different from other falling patients. For example, those with hip fractures caused by falls [20, 21] show a higher degree of dependence prior to the fracture. Also, patients of the same age and sex who have not suffered fractures caused by falls, have a lower prevalence of comorbidity and polypharmacy [22].

The results show that most elderly patients that sustained a wrist fracture after a traumatic fall completely recovered their functionality, based on their BADL and IADL performance reported six months after the traumatic event. Nevertheless, wrist fractures have a notable impact on the overall functional capacity of a third (33 %) of patients. Considering BADL and IADL separately the impact is still remarkable with a 24 and 23 % of patients presenting losses respectively. And this impact is still evident six months after the accidental fall that caused the fracture. The magnitude of the impact of wrist fracture has been measured in terms of functional and HRQoL decline and persistence of pain [23–25]. The occurrence of this traumatic event transforms the daily lives of these patients, shifting them from autonomy towards disability and dependence. Indeed, it has been hypothesized [26] that wrist fractures may be a trigger for progressive functional decline.

According to our findings, significant baseline differences may be found among fractured patients when their functional decline (basic or instrumental) is assessed six months after the fracture. Functional recovery (improvement or maintenance of previous BADL or IADL performance, versus deterioration, as defined in the methods section) is associated with a series of factors related to the patient, both in the health status sphere (comorbidity, polypharmacy, severe chronic conditions and low HRQoL) and the social sphere (lower educational level and social support), and also to the type of treatment (surgery and lack of referral for rehabilitation). Additionally, patients presenting decline in BADL, but not in IADL had a history of repeated falls. These results are consistent with previously published articles where functional outcome after a wrist fracture has shown to be associated with age, educational level and treatment procedure [4, 27–29].

The described characteristics of patients with BADL functional decline match the definition of a frail patient [30, 31]. Frail patients are independent but with decreased functional reserve. This condition makes it easier for an acute event to initiate a progressive and continuous decline to dependence, hospitalization and even death.

The derived multivariate models reinforce these results in the sense that patients with BADL functional decline, meaning those that are dependent six months after the fall, have a profile consistent with frailty at the time of the fracture, with advanced age, severe chronic diseases, low functional performance, and repeated episodes of accidental falls with previous fractures.

Our findings highlight the impact of wrist fractures on functional impairment and the generation of dependence. Further, they underline the role of two key factors that regulate the aforementioned impact: the baseline functional situation and the clinical characteristics of the patient. In order to prevent the functional decline associated to wrist fractures, these two factors should be considered during the first contact with the fractured patient. There is a need for, on the one hand, the assessment of functional capacity prior to the fracture, as a key factor in the decision making process of treatment and rehabilitation provision, and on the other, the identification of frail patients, in order to provide them with a comprehensive answer to their complex needs.

This study has some limitations, the most relevant being that prefall functional and health status were obtained from the patient, retrospectively. This information was collected as soon as possible and through standardized instruments in order to minimize the recall effect. The way information was obtained may also constitute a limitation given that the instruments were applied by interviewers at baseline and autocompleted at follow. The used instruments were suitable for both types of administration. An additional limitation is related to the loss to follow-up of individuals over time, though our response rate (74 %) can be considered acceptable [32]. Finally, it should be underlined that not all the functional deterioration observed in these participants is necessarily attributable to the fracture and no data are presented for a control group given that the main focus of this study was to characterize factors associated with the actual loss of function after the fall and fracture.

## Conclusions

Wrist fractures need to be considered as a relevant factor in the pathway of dependence. Awareness of the profile of patients with high risk of poor functional recovery may constitute a first step in the generation of comprehensive protocols for wrist fractures that would prevent some of the burden of dependence generated by them.
